# Cardiac magnetic resonance (CMR) and three dimensional echocardiography (3DE) in complete evaluation of Ebstein anomaly

**DOI:** 10.1186/1532-429X-14-S1-P109

**Published:** 2012-02-01

**Authors:** Gurpreet S Gulati, Pranab Kumar, Sivasubramanian Ramakrishnan, Priya Jagia, Sanjiv Sharma

**Affiliations:** 1 Cardiovascular Radiology, All India Institute of Medical Sciences, Delhi, India; 2 Cardiology, All India Institute of Medical Sciences, Delhi, India

## Summary

This study on 10 patients highlights the complementary role of Cardiac magnetic resonance (CMR) and 3D echocardiography in Ebstein anomaly. While 3D echocardiography is excellent for depicting valve anatomy, CMR assesses the functional RV, presence of fibrosis and PA anatomy. GOSE score is accurately calculated on CMR.

## Background

Two dimensional echocardiography (2DE) may be inadequate in detailed assessment of Ebstein anomaly. Complementary role of 3DE and CMR in this anomaly is not well defined in literature.

## Methods

A comparative, non-randomized cohort study of 10 patients with 2DE diagnosis of Ebstein anomaly was carried out after written informed consent and approval from Institute’s Ethics Committee. All patients underwent 3DE and CMR. Patients with congestive heart failure and contraindications to MRI were excluded. 3DE data was obtained on Philips iE33 (Bothell, WA, USA). CMR was done with 1.5T (Siemens, Avanto, Germany) with SSFP (steady state free precession) single shot, SSFP cine and delayed enhanced (DE) sequences in axial and cardiac imaging planes. Imaging parameters included: Diameter and volume of RA, volume of atrialized RV, volume and function of residual RV, tethering and mobility of anterior leaflet (AL), presence and degree of displacement of posterior (PL) and septal (SL) leaflets, any DE, Carpentier classification and GOSE score. 3DE and CMR data were compared with appropriate statistical techniques.

## Results

Mean patient age was 24 years (range 8-45); there were five females. All patients had ASD and severe tricuspid regurgitation. While SL was displaced in all, PL was displaced in eight and absent in one, similarly seen on both CMR and 3DE. The absent PL was actually seen to be plastered to RV wall on CMR. While CMR could show a free or tethered AL, detailed assessment (points of tethering/ bubbling and severity of restriction) was only possible on 3DE. While there was significant correlation between 3DE and CMR values of residual RV end diastolic volume, atrialized RV volume and SL displacement, this was not significant for RV end systolic volume and ejection fraction (p=0.6). The Carpentier type matched on both techniques. CMR additionally showed adequate sized pulmonary arteries (PA) in all, directly measured residual RV volume and function (small in 5, all Carpentier type III/IV) and showed presence (6) or absence (4) of DE. DE was along SL alone in three (type III) or also along septum with/without AL in three (type IV). The CMR GOSE score averaged 1.29 (range 0.7-1.88).

## Conclusions

In Ebstein anomaly, while 3DE provides detailed assessment of AL, CMR accurately quantifies the residual RV, GOSE score, and demonstrate fibrosis and PA anatomy. Both are complementary in planning surgical repair.

## Funding

NIL.

